# Virtual crossmatching reveals upregulation of placental HLA-Class II in chronic histiocytic intervillositis

**DOI:** 10.1038/s41598-024-69315-5

**Published:** 2024-08-12

**Authors:** Chloe A. Brady, Laura B. Ford, Chloe Moss, Zhiyong Zou, Ian P. Crocker, Alexander E. P. Heazell

**Affiliations:** 1 Tommy’s Maternal and Fetal Health Research Centre, St Mary’s Hospital, The University of Manchester, Manchester, UK; 2 Transplantation Laboratory, Manchester Royal Infirmary, Manchester University NHS Foundation Trust, Manchester, UK; 3 Saint Mary’s Hospital, Manchester University NHS Foundation Trust, Manchester, UK

**Keywords:** Transplant immunology, Reproductive disorders, Inflammatory diseases

## Abstract

Chronic histiocytic intervillositis (CHI) is a recurrent placental lesion where maternal macrophages infiltrate the intervillous space. Its cause is unknown, though due to similarities to rejected allografts one hypothesis is that CHI represents maternal–fetal rejection. Here, virtual crossmatching was applied to healthy pregnancies and those with a history of CHI. Anti-HLA antibodies, measured by Luminex, were present in slightly more controls than CHI (8/17 (47.1%) vs 5/14 (35.7%)), but there was no significant difference in levels of sensitisation or fetal specific antibodies. Quantification of immunohistochemical staining for HLA-Class II was increased in syncytiotrophoblast of placentas with CHI (Grade 0.44 [IQR 0.1–0.7]) compared to healthy controls (0.06 [IQR 0–0.2]) and subsequent pregnancies (0.13 [IQR 0–0.3]) (*P* = 0.0004*).* HLA-Class II expression was positively related both to the severity of CHI (r = 0.67) and C4d deposition (r = 0.48). There was no difference in overall C4d and HLA-Class I immunostaining. Though increased anti-HLA antibodies were not evident in CHI, increased expression of HLA-Class II at the maternal–fetal interface suggests that they may be relevant in its pathogenesis. Further investigation of antibodies immediately after diagnosis is warranted in a larger cohort of CHI cases to better understand the role of HLA in its pathophysiology.

## Introduction

Throughout healthy human pregnancy the fetus is tolerated by the maternal immune system despite possessing genetically foreign paternal human leukocyte antigen (HLA). This ability of the semi-allogeneic fetus to evade mounting an inflammatory response has led the conceptus to be coined as the ‘most successful graft’^1^. In pregnancy, there are several mechanisms which promote maternal–fetal tolerance, including specialised placental HLA expression and maternal immune cell adaptations^2–5^. The failure of these tolerogenic processes has been linked to the pathogenesis of multiple inflammatory placental disorders resulting in adverse pregnancy outcomes such as preterm birth, miscarriage and stillbirth^6–8^.

One example of a placental lesion with an immune etiology is chronic histiocytic intervillositis (CHI), in which maternal macrophages infiltrate the intervillous space^9^. CHI is associated with fetal growth restriction (FGR), and in severe cases can result in miscarriage or stillbirth^10^. Though the cause of CHI is unknown, previous case reports and series have proposed an antibody-mediated component, given its increased incidence in women with autoimmune disease^11,12^, high recurrence rate^13,14^, evidence of increased anti-HLA antibodies^15,16^, and in select cases deposition of complement split product C4d^15,17^. In addition, some cases of CHI also exhibit accompanying fibrin deposition^10^ and anti-paternal T cells have been detected in several women with the disorder^16^. As many of these pathological features are also common to rejected allografts, CHI is hypothesised to represent maternal–fetal rejection and is consequently treated with immunosuppressive medication^12,18–20^. Despite suggestions of antibody involvement from case reports, CHI’s rarity (~ 0.17% of pregnancies^10^) has made conducting larger scale studies into its pathophysiology difficult. Research into the condition is further complicated by the fact its diagnosis can only be made after delivery of the placenta, in most cases when the health or survival of the fetus has already been compromised.

For decades, predicting risk of rejection in recipients of solid organ transplants has been possible via laboratory crossmatching techniques which informs clinical management and facilitates maintenance of a foreign graft for a sustained period. In this study, we aimed to apply virtual crossmatching techniques to cases of CHI to investigate the possibility of placental rejection and characterise the HLA expression profile in inflamed placentas. In doing so, we hypothesised that affected women would exhibit increased evidence of humoral involvement including high titre anti-HLA antibodies and deposition of C4d, similar to that observed in the rejection of a transplanted organ.

## Materials and methods

### Participant recruitment and sample collection

Participants were retrospectively identified by searching medical records at Saint Mary’s Hospital, Manchester, UK, for women with a diagnosis of CHI in a previous pregnancy. Cases of CHI with archived placental tissue available for analysis from the Paediatric and Perinatal Histopathology Department were included. Placental tissue from cases of CHI and healthy controls were received as formalin-fixed paraffin embedded (FFPE) blocks, and accompanying hospital records collected where available, detailing maternal demographic data and obstetric history. For certain participants, accompanying medical history could not be retrieved for the study as they had delivered at another centre or records were unavailable at the time of retrieval. These cases were excluded from statistical analysis of demographic characteristics and pregnancy outcome, and analysis was restricted to blood and/or placental tissue. An initial subset of FFPE placental tissue from healthy pregnancies was provided for the study by the Paediatric and Perinatal Histopathology Department, for which matched maternal blood was not collected. Pregnancy outcomes consisted of livebirth, miscarriage (fetal death < 24 weeks’ gestation), stillbirth (fetal death ≥ 24 weeks’ gestation) and termination of pregnancy (TOP). Individualised birthweight centiles were calculated using the GROW centile calculator for pregnancies ≥ 20 weeks’ gestation^21^.

Blood was collected prospectively both from women in their second or third trimester attending Tommy’s Rainbow Clinic for care in a subsequent pregnancy following a previous diagnosis of CHI, and healthy control pregnancies on the date of delivery at Saint Mary’s Hospital. Vacutainer^™^ EDTA samples were obtained and centrifuged at 2000* g* for 10 min at 4 °C, and plasma supernatant retrieved and stored as aliquots at − 80 °C. The buffy coat layer was also retrieved at the red cell/plasma interface for maternal DNA analysis and stored in the same manner. Where participants were giving birth at Saint Mary’s Hospital, placental tissue was collected and fixed in formalin before embedding in paraffin wax to produce FFPE blocks. Umbilical cord tissue was also sampled, rinsed and frozen at − 80 °C for later use.

Informed and written consent was obtained from all study participants. For archived tissue from Paediatric and Perinatal Histopathology, ethical approval was granted by NRES Committee London-City & East (REC ref: 14/LO/1352). For samples gathered before July 2018, approval was granted by NRES Committee Northwest-Greater Manchester West (14/NW/1149). Between February and July 2018, the NRES Committee South East Coast-Surrey Research Ethics Committee (16/LO/1666), and samples collected after this period were obtained under the Tommy’s Project Ethics (15/NW/0829). All methods were performed in accordance with ethical committee guidelines and the Declaration of Helsinki.

### Immunohistochemistry and semi-quantitative analysis

Immunostaining for C4d in healthy control placentas and those from index cases of CHI and subsequent pregnancies was carried out by the Department of Adult Histopathology, Manchester University NHS Foundation Trust. 5 µm sections of FFPE tissue were cut from the centre and edge of the placenta, mounted onto SuperFrost slides (ThermoFisher Scientific) and dried overnight at 38 °C before long-term storage at room temperature. A tissue biopsy section from a rejected kidney with confirmed antibody-mediated rejection was provided by Manchester Royal Infirmary Transplantation Laboratory (MRITL), for use as a positive control. Slides were deparaffinised in EZ Prep Volume Adjust (Ventana Co., Arizona, USA) according to manufacturer’s instructions and washed in TRIS-based Reaction Buffer (pH 7.6). Antigen retrieval was achieved using heat and TRIS–EDTA-boric acid (pH 8.4, Ventana Co.) for 60 min. Ultraviolet inhibitor blocking solution was applied for 4 min before a further 30-min incubation at room temperature with rabbit polyclonal anti-human C4d antibody (Cell Marque, Sigma Aldrich, Missouri, USA, 1:75 dilution). Slides were then incubated in horseradish peroxidase-linked secondary antibody for 8 min before an 8-min incubation in diaminobenzidine chromogen. To amplify positive staining, a copper enhancer was applied for 4 min, before a 12-min counterstain in Haematoxylin II and 4 min in bluing reagent. Finally, slides were dehydrated by sequential washes in solutions of 70% (2 × 3 min), 95% (2 × 3 min) and 100% industrial methylated spirit (ThermoFisher) (3 × 3 min) before 1 × 2 min and 2 × 10 min immersions in Histoclear (Thermo Fisher). After staining, placental slides were mounted with DPX and coverslips and left to dry.

Immunohistochemical staining for HLA-Class I and II in placental tissue was undertaken at the University of Manchester using a Leica BOND RX Research Stainer. Slides were cut and prepared as previously described, dewaxed and heated in citrate buffer for 30 min in Leica BOND standard solutions for antigen retrieval. Endogenous peroxidase was quenched using 3% (v/v) hydrogen peroxide (VWR International, Pennsylvania, USA) for 10 min at room temperature. Following 2 × 5 min washes in distilled water, blocking of non-specific antibody binding was conducted using 10% (v/v) normal goat serum (Sigma-Aldrich) in 0.1% TBS-Tween (Biotium, UK) for 30 min at room temperature. Sections were then incubated for one hour at 37 °C with mouse monoclonal anti-human HLA Class I (Abcam, 5 μg/ml) or Class II (Santa Cruz Biotechnology, Texas, USA 5 μg/ml) diluted in blocking solution. After a 5 min wash in distilled water, 2 × 5 min washes in Leica wash solution and another 5 min in distilled water, sections were incubated with biotinylated polyclonal goat anti-mouse secondary antibody (AAT Bioquest, California, USA 3.3 µg/ml) for 30 min at room temperature followed by washes in distilled water and wash solution as previously described. Avidin-peroxidase (Sigma-Aldrich, 5 µg/ml in TBS) was applied for 30 min at room temperature to amplify the secondary antibody signal before 2 × 5 min washes in wash solution and 1 × 5 min wash in distilled water. Diaminobenzidine (DAB) chromogen (BD Biosciences, New Jersey, USA) was reconstituted according to manufacturer’s instructions and dropped onto each section for secondary antibody detection. Following an 8-min incubation and 5-min wash in distilled water, placental sections were finally incubated (2 s) in haematoxylin as a nuclear counterstain. Slides were then left in warm water for 5 min and then transferred into cold water. Dehydration of sections was conducted by sequential incubation in solutions of 70% (2 × 3 min), 95% (2 × 3 min) and 100% IMS (3 × 3 min) before 1 × 2 min and 2 × 10 min incubations in Histoclear (National Diagnostics, Georgia, USA). DPX mounting medium was dropped onto each section before coverslip application and storage. Negative sections utilised to ensure antibody specificity were treated identically except for omission of primary antibody which was replaced with non-immune mouse IgG (Sigma-Aldrich) at the same working concentration. Spleen tissue was used as a positive control for both HLA-Class I and HLA-Class II antibodies. C4d and HLA Class I and II slides were scanned at the University of Manchester Bioimaging Facility using brightfield microscopy and a 3D Histech Panoramic 250 Flash Slide Scanner.

Grading of C4d deposition and HLA expression was carried out by two separate reviewers blinded to sample identity. Scanned slides were opened using QuPath Version 0.3.0 RRID:SCR_018257 computerised image analysis software^22^ and a 1mm^2^ grid superimposed upon the scanned slide image. A random number generator was then used to select eight regions for blinded semi-quantitative analysis of staining intensity. Intervillous C4d and HLA positivity was graded on a scale of 0 to 3; 0 = 0% to 5% of villi affected; 1 = 5% to 25%; 2 = 25% to 75%; and 3 ≥ 75% as previously described^17^. Only positive staining along the apical surface of villi which was in contact with maternal blood was considered in the analysis. C4d and HLA scores from the centre and edge placental regions were averaged to provide a measure for each placenta, before both separate reviewer’s scores were averaged to give an overall score.

### Anti-HLA antibody screening of maternal plasma

For detection of class I and II anti-HLA antibodies, maternal EDTA plasma samples were processed by MRITL. A mixed Luminex assay was used to initially determine any presence of anti-HLA antibodies, consisting of LABScreen Mixed Beads (One Lambda Inc, Thermo Fisher) coated in several HLA antigens and each possessing a unique combination of fluorochromes. Maternal plasma was incubated with the mixed beads at 22 °C for 15 min before washing in LABScreen wash buffer according to manufacturer’s protocol. R-phycoerythrin (PE)-conjugated goat anti-human IgG and PE-conjugated donkey anti-human IgM secondary antibodies were then added for 5 min at 22 °C to allow detection of bound antibody. After a final wash in wash buffer, beads were resuspended in buffer before transfer to a 96-well PCR plate for Luminex LabScan3D analysis using a dual laser system to identify the presence of antibody in patient plasma binding to beads, giving a measurement of mean fluorescence intensity (MFI). Data were inputted into HLA Fusion One Lambda Software and checked with established criteria from the MRITL for detection of false positives or negatives. Samples testing positive were then retested in the same manner but with specific LABScreen beads possessing only a single antigen for identification of anti-HLA antibody specificity. Antibodies with an MFI value of 2000 or more were considered positive in accordance with MRITL criteria.

### Maternal and fetal HLA genotyping

In the case of a positive anti-HLA antibody screen, maternal and fetal DNA (or maternal DNA alone where fetal tissue was not available) were extracted for determination of HLA genotype from thawed EDTA buffy coat and snap frozen umbilical cord samples, respectively. Samples were processed using the DNeasy Blood & Tissue Kit (Qiagen, Germany) according to kit instructions.

Extracted DNA samples were analysed at the MRITL to determine HLA genotype via the Luminex LABType^™^ Sequence Specific Oligonucleotide (SSO) HLA Typing protocol to fluorescently tag and identify HLA alleles. Results were reviewed by a State Registered Clinical Scientist (LBF) to confirm HLA genotype and establish any false positive bead reactions. The number of fetal-specific anti-HLA antibodies (FSAs) and their MFI was then noted in positive cases where fetal DNA was available.

### Percentage calculated reaction frequency

Using maternal anti-HLA antibody specificities and HLA genotype, percentage calculated Reaction Frequency (%cRF) was established for each participant testing positive via input of the results of Luminex screen into the NHS Blood and Transplant Kidney %cRF tool^23^. %cRF reflects the percentage of donors anticipated to have an unsuitable HLA profile in the context of renal transplantation and is based on the HLA type from 10,000 donors, whose HLA type reflects those of the UK general population. Organ recipients with no HLA incompatible donors are assigned a %cRF of 0% and are unsensitised with 100% of donors being suitable, whereas those with a score of > 80% are highly sensitised and prove more challenging to find a suitable donor, as > 80% of donors are considered unsuitable.

### Prediction of crossmatch results

In participants with FSAs, results of T and B cell flow cytometry crossmatch (FXCM) and complement-dependent cytotoxicity (CDC) crossmatch were predicted using a formula developed by the MRITL^24^. HLA specificities and respective FSA MFI values were inputted to give an estimation of positive or negative result. Where results were in between the in-house cut-off values for a positive or negative result, these were described as equivocal, and could not be reliably predicted.

### Statistical analysis

All statistical analysis and creation of graphs was undertaken using GraphPad Prism v9 (GraphPad Software, USA). Normality was determined by the Shapiro–Wilk test. Continuous demographic data were analysed using ordinary one-way ANOVA with Dunn’s multiple comparisons test or Kruskal–Wallis test for normally distributed and non-normally distributed data, respectively. Interobserver agreement of C4d and HLA staining was determined by calculation of the weighted Kappa between reviewer scores and grading was analysed via Kruskal–Wallis test with Dunn’s multiple comparisons where this was significant. Categorical demographic data and proportions of antibody-positive participants were analysed using Fisher’s exact test. For %cRF values the Mann–Whitney test was run to determine statistical differences. Statistical significance was set at *P* < 0.05 for each test. To relate HLA-Class I and II expression with CHI severity, scores were compared to CD68^+^ counts previously carried out on placentas from index cases of CHI^18^

## Results

### Participant demographic characteristics

Seventeen index cases of CHI had available placental tissue as FFPE embedded blocks, with eight healthy controls provided by the Department of Paediatric Histopathology. A further nineteen healthy controls and fifteen women in subsequent pregnancies after CHI were recruited to the study. For two index cases, demographic information and pregnancy outcomes could not be obtained as records were unavailable or participants received their care at another centre. Maternal demographic and pregnancy outcome data corresponding to one subsequent pregnancy was limited as the participant chose to give birth at another hospital. Despite this, these pregnancies were included in the study due to availability of placental tissue and the rarity of CHI. An overview of recruitment for the study is provided in Supplementary Fig. 1. Demographic characteristics of remaining study participants and pregnancy outcomes are shown in Table 1. There were no significant differences in maternal age, BMI, ethnicity or pregnancies conceived via oocyte donor. Gravidity and parity were significantly higher in index pregnancies compared to controls*.* In women returning for care in a subsequent pregnancy following diagnosis, parity was significantly lower compared to index cases of CHI. Women returning for care in subsequent pregnancies had a higher incidence of autoimmune disease compared to controls (*P* = 0.012). Adverse outcome was more common in index pregnancies with CHI compared to control (*P* < 0.0001) and subsequent pregnancies (*P* < 0.0001). Rates of Caesarean section were higher in control pregnancies compared to index cases of CHI (*P* = 0.0003), and gestation at delivery was decreased in index (*P* = 0.0031) and subsequent pregnancies (*P* = 0.016). Individualised birthweight centile was significantly decreased in index cases of CHI compared to control (*P* < 0.0001) and subsequent pregnancies (*P* = 0.016*)*. There was a higher proportion of male fetuses in subsequent pregnancies compared to controls (*P* = 0.02*).*

**Table 1:** Participant demographic characteristics of healthy control pregnancies, index pregnancies with chronic histiocytic intervillositis (CHI) and subsequent pregnancies of women with a previous diagnosis of CHI.

	Control	Index CHI	Subsequent pregnancy	*P* value
*N*	27	15	15	
Maternal age (years)	32.9 (± 4.7)	29.5 (± 6.1)	30.6 (± 6.3)	0.15
Maternal BMI	25.9 (± 4.5)	27.9 (± 4.3)	26.4 (± 5.4)	0.52
Unknown	0	5	0	
Ethnicity				0.54
Asian	3 (11.1%)	3 (21.4%)	5 (33.3%)	
Black African	4 (14,8%)	1 (7.1%)	0	
Eastern European	1 (3.7%)	0	1 (6.7%)	
White British	18 (66.7%)	10 (71.4%)	9 (60.0%)	
White Irish	1 (3.7%)	0	0	
Unknown	0	1	0	
Gravidity	3 (1–11)	4 (2–10)	3 (2–8)	0.01
Parity	2 (0–8)	3 (1–7)	1 (0–3)	0.001
Maternal autoimmune disease	0	2 (13.3%)	4 (26.7%)	0.015
Pregnancy outcomes				< 0.0001
Livebirth	27 (100%)	2 (12.5%)	13 (92.9%)	
Miscarriage	0	1 (6.3%)	1 (7.1%)	
TOP	0	1 (6.3%)	0	
Stillbirth	0	12 (75.0%)	0	
Unknown	0	0	1	
Oocyte donor pregnancy	0	0	1 (6.7%)	0.53
Caesarean section (> 24 weeks)	20 (74.1%)	2 (14.3%)	7 (46.7%)	0.0006
Gestation at delivery (weeks)	39 (35–41)	33.5 (23–40)	37 (17–39)	0.001
Birthweight (g)	3424 (2530–4355)	1289 (470–3370)	3084 (785–4072)	< 0.0001
Unknown	0	1	2	
IB﻿C	63.6 (11–95.2)	4.4 (0–45.3)	33.2 (0.8–99.9)	< 0.0001
Unknown	0	1	2	
Fetal sex (*n* males)	12 (44.4%)	7 (50.0%)	11 (73.3%)	0.048
Unknown	0	1	2	
CHI in placenta	N/A	15 (100%)	0 (100%)*	N/A
No histopathology report	N/A	0	1	

Index pregnancy refers to a participant’s first pregnancy to be diagnosed with CHI. Normally and non-normally distributed variables are expressed as median (range) and mean (± standard deviation) respectively. Categorical variables are shown as *N* (percentage). Miscarriage and stillbirth were defined as fetal death below or above 24 weeks’ gestation, respectively.

*BMI* body mass index, *IBC* individualised birthweight centile, *TOP* termination of pregnancy.

*Placental tissue was sent for histopathological examination in ten of fifteen subsequent pregnancies.

By definition, all index cases had a diagnosis of CHI listed on their placental histopathology report. Placental tissue was sent for histopathological examination in ten subsequent pregnancies. There was no histopathological diagnosis of recurrent CHI noted in any subsequent pregnancies. Eleven of fifteen (73.3%) subsequent pregnancies were medicated with aspirin, low molecular weight heparin, hydroxychloroquine and prednisolone. The remaining four (26.7%) subsequent pregnancies were treated with aspirin, low-molecular-weight heparin and hydroxychloroquine only.

### C4d deposition in index cases of CHI

**Figure 1:**
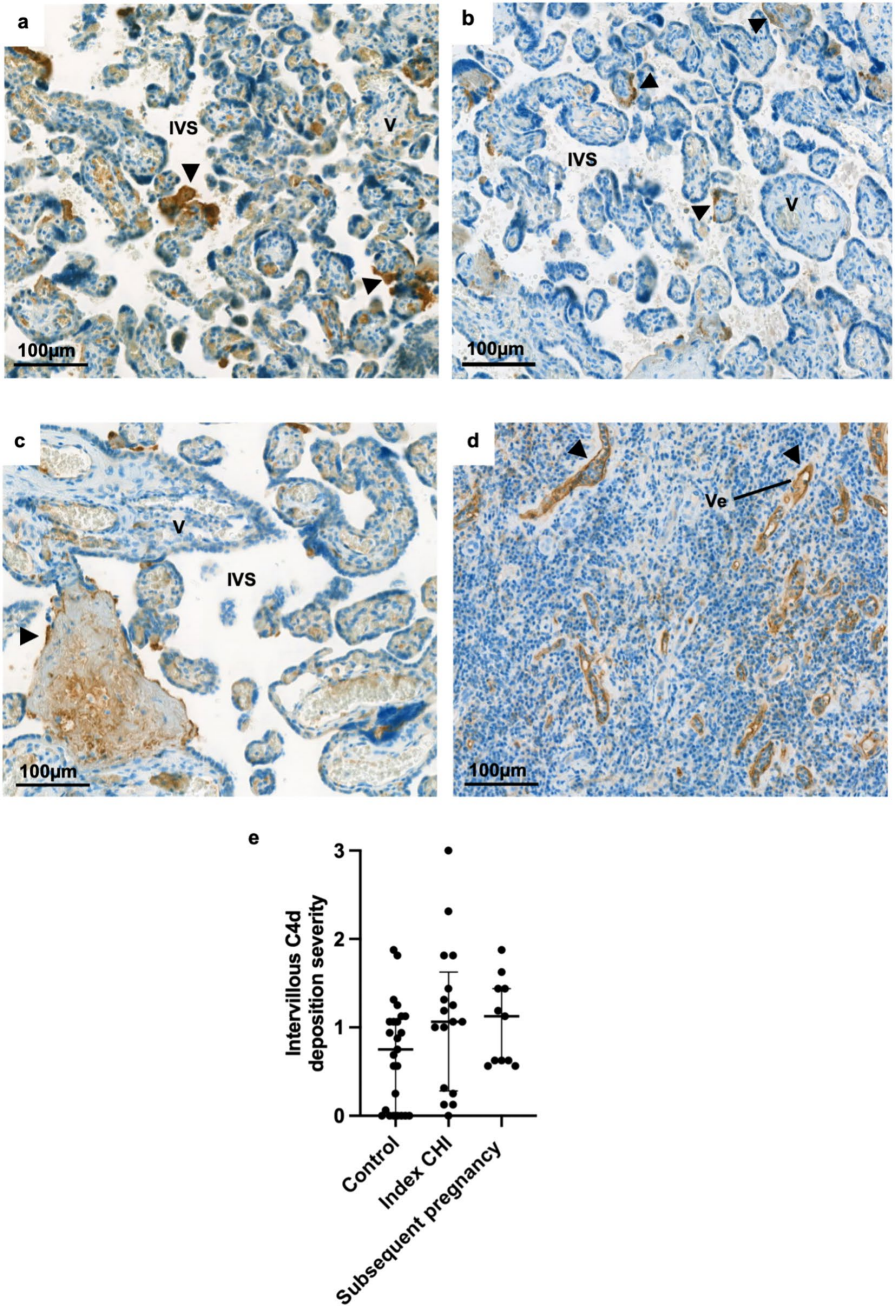
Complement split product C4d in placental tissue and a kidney graft with confirmed antibody mediated rejection (AMR). (**a**) Healthy control placenta. (**b**) Index case of chronic histiocytic intervillositis (CHI). (**c**) Subsequent pregnancy following CHI diagnosis. d) A kidney biopsy with antibody mediated rejection (AMR). (**e**) Semi-quantitative grading of C4d staining. Bars represent median and error bars interquartile range. Statistical significance was tested using Kruskal–Wallis test. *IVS* intervillous space, *V* villi, *Ve* vessel. Arrows depict positive staining.

FFPE tissue was available for analysis in pregnancies from twenty-five healthy controls, seventeen index cases of CHI and eleven subsequent pregnancies. C4d was apparent in healthy control placentas (Fig. 1a), those from index cases of CHI (Fig. 1b) and subsequent pregnancies (Fig. 1c). C4d localised along the apical surface of the syncytiotrophoblast, appearing similar to the vessels of a rejected kidney (Fig. 1d). Weighted Kappa for interobserver agreement was calculated at 0.43, indicating moderate agreement. Amongst all three groups, the degree of C4d deposition varied widely between cases, there was no significant difference between the levels of C4d deposition in index CHI (median 1.06 [IQR 0.3–1.6]) and subsequent pregnancies (1.13 [IQR 0.6–1.4]) compared to controls (0.75 [IQR 0–1.1]) (Fig. 1e). The extent of C4d deposition was compared to the degree of macrophage infiltration previously quantified in these cases, which showed no relationship (r = -0.001, *P* = 0.99).

### Expression of HLA-class I and II at the maternal–fetal interface in CHI

**Figure 2:**
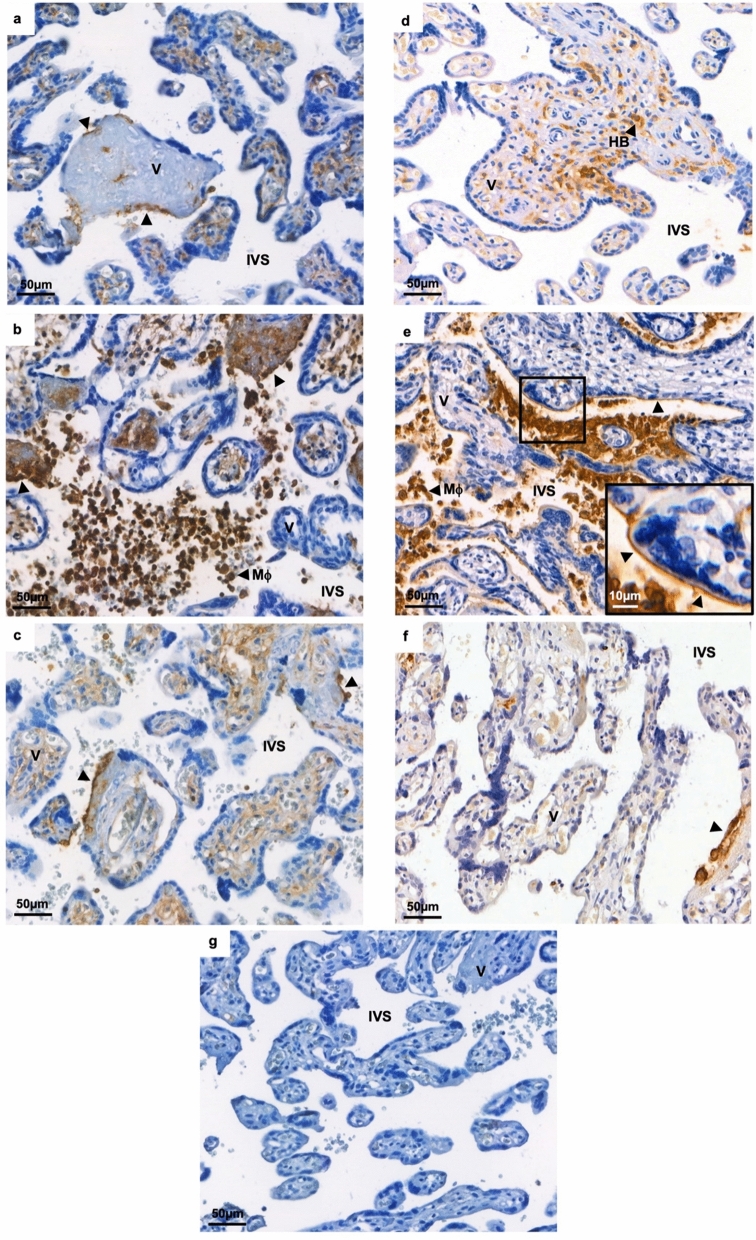
HLA expression in placenta. HLA-Class I immunohistochemical staining in (**a**) a healthy control placenta, (**b**) an index case of chronic histiocytic intervillositis (CHI) and (**c**) a subsequent pregnancy following diagnosis and treatment. HLA-Class II immunohistochemical staining is shown in (**d**) a healthy control placenta, (**e**) an index case of CHI and (**f**) a subsequent pregnancy. (**f**) IgG isotype control. *HB* Hofbauer cell, *IVS* intervillous space, *M* macrophage, *V* villus. Arrows depict positive staining.

HLA-Class I expression was low within placentas of healthy controls, index cases of CHI and subsequent pregnancies, with only a small degree of HLA-Class I expression on the surface of certain villi in areas where the syncytiotrophoblast layer appeared denuded (Fig. 2a-c). In index cases of CHI, the majority of positive HLA-Class I staining was observed in infiltrating maternal macrophages, however there were occasional focal areas of expression around what appeared to be necrotic villi and areas of fibrin deposition (Fig. 2b). HLA-Class II was rarely observed at the maternal–fetal interface in healthy control pregnancies and was instead mainly found in Hofbauer cells within fetal villi (Fig. 2d). In contrast, index cases of CHI demonstrated linear positive staining for HLA-Class II on the apical surface of certain areas of syncytiotrophoblast (Fig. 2e). This staining was often in proximity to areas of macrophage infiltration. Most placentas of subsequent pregnancies closely resembled healthy controls, with only sparse areas of HLA-Class II in contact with the intervillous space (Fig. 2f).

**Figure 3:**
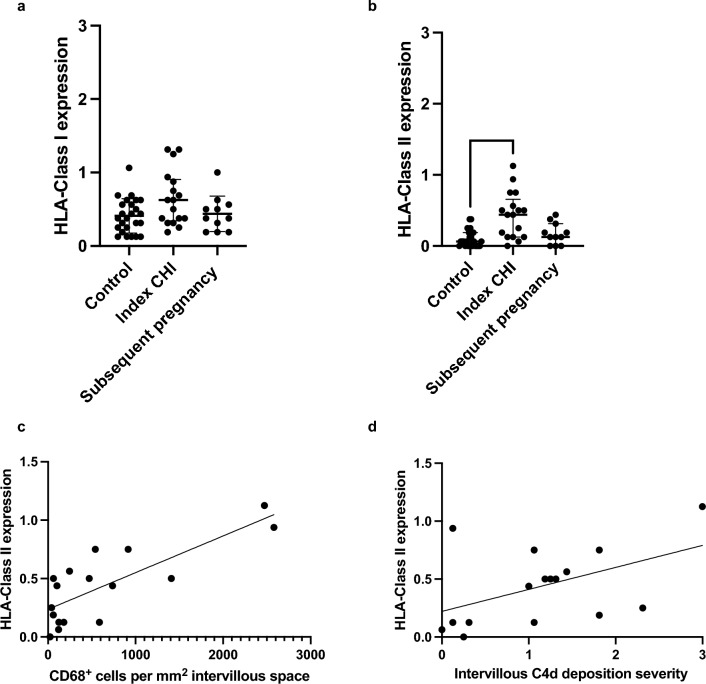
Semi-quantitative grading of (**a**) HLA-Class I and (**b**) Class II expression in placentas from healthy control pregnancies, index cases of chronic histiocytic intervillositis (CHI) and subsequent pregnancies following diagnosis and treatment. Bars represent median and error bars interquartile range. Statistical significance was assessed via Kruskal–Wallis test, with Dunn’s multiple comparisons where differences were found. (**c**) and (**d**) Relationship between CD68 + macrophage infiltration in index cases of CHI and HLA-Class II and C4d deposition as determined by Spearman’s rank and simple linear regression.

The weighted Kappa for grading of HLA expression along the apical surface of the syncytiotrophoblast was 0.36, indicating fair agreement. No significant difference was found in average HLA-Class I expression between healthy control pregnancies (median 0.38 [IQR 0.2–0.6]), index cases of CHI (0.63 [IQR 0.3–0.9]) and subsequent pregnancies (0.38 [IQR 0.2–0.6]) (Fig. 3a). Average HLA-Class II expression was significantly increased in index cases of CHI (0.44 [IQR 0.1–0.7]) compared to healthy controls (0.06 [IQR 0–0.2]) (*P* = 0.0002), but not subsequent pregnancies (0.13 [IQR 0–0.3]) (Fig. 3b). Syncytiotrophoblast HLA-Class II expression in index cases of CHI demonstrated a positive relationship both with the extent of intervillous CD68^+^ macrophage infiltration (r = 0.67, *P* = 0.004) (Fig. 3c) and C4d deposition (r = 0.48, *P* = *0.05*) (Fig. 3d).

### Maternal anti-HLA antibody screening

**Figure 4:**
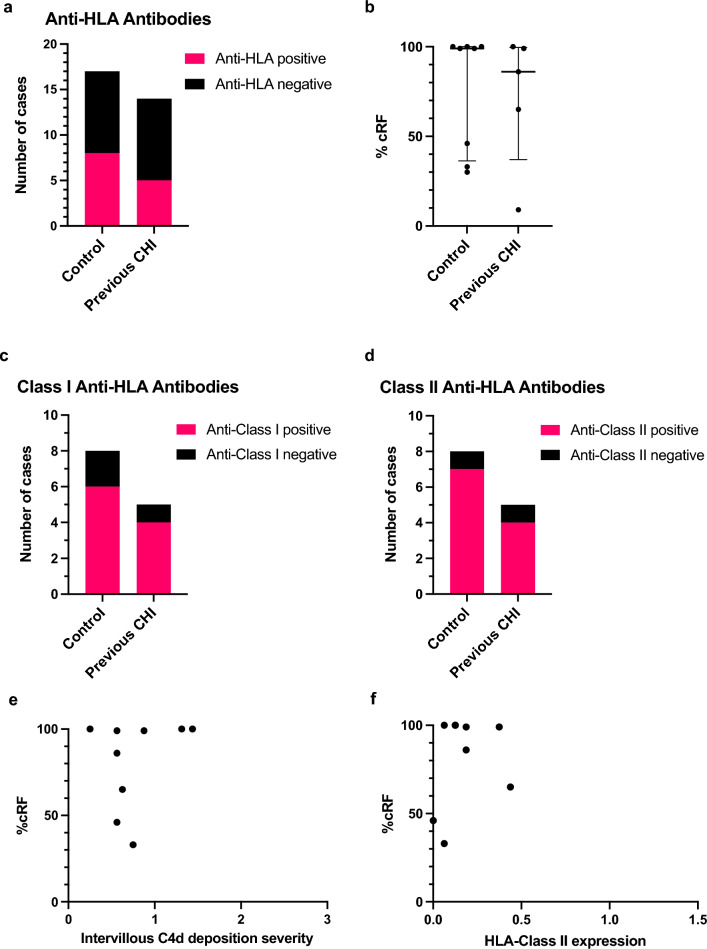
Anti-HLA antibodies in healthy control pregnancies and subsequent pregnancies of women with a previous diagnosis of chronic histiocytic intervillositis (CHI). (**a**) Frequency of anti-HLA antibody positivity determined via Luminex screening. (**b**) Percentage calculated reaction frequency (%cRF) of antibody-positive study participants. %cRF reflects the percentage of donors an individual would be likely to reject based on the general population. Bars represent median and error bars interquartile range. (**c**) Class I Anti-HLA (HLA-A, B and C) antibody positivity. (**d**) Class II Anti-HLA (HLA-DP, DQ, DR) antibody positivity. e) %cRF values of antibody-positive participants versus placental intervillous C4d staining.

Seventeen controls and fourteen participants with previous CHI had plasma available for analysis by Luminex for the presence of anti-HLA antibodies. There was no statistically significant difference in the proportion of healthy control participants tested positive for anti-HLA antibodies (Class I and II) compared to those with a history of CHI (8/17 (47.1%) vs 5/14 (35.7%), respectively, Fig. 4a). There was also no significant difference in the median level of sensitisation between antibody positive healthy controls and women with a diagnosis of CHI (99.0 [interquartile range (IQR) 36.3–100] vs 86.0 [IQR 37.0–99.5], respectively) (Fig. 4b). Five of eight (62.5%) anti-HLA antibody positive controls were classed as highly sensitised with a %cRF > 80%, compared to three out of five (60.0%) participants with previous CHI.

Amongst anti-HLA positive participants, a similar proportion of those with a history of CHI displayed Class I positivity compared to controls (4/5 (80.0%) vs 6/8 (75.0%), respectively) (Fig. 4c). Four of five (80.0%) participants with CHI tested positive for Class II anti-HLA antibodies compared to seven of eight controls (87.5%) (Fig. 4d). These variations in proportions were not statistically significant. Six antibody positive control participants and three participants with previous CHI had matched placental tissue available for analysis. Amongst these who tested positive for anti-HLA antibodies, %cRF was not related to the severity of intervillous C4d deposition or HLA-Class II expression (Fig. 4e and f, respectively).

### Screening for fetal-specific antibodies

**Figure 5:**
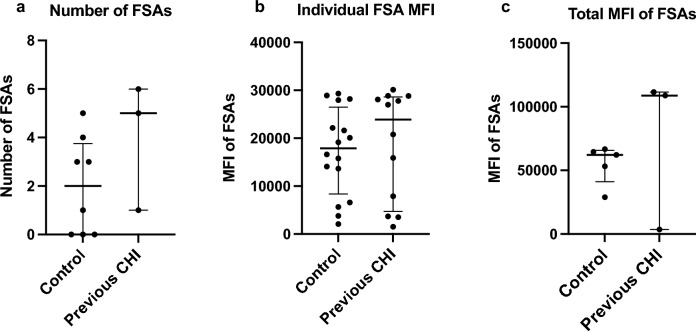
Fetal-specific anti-HLA antibodies (FSAs) in healthy control pregnancies and subsequent pregnancies of women with a previous diagnosis of chronic histiocytic intervillositis (CHI). (**a**) Number of FSAs determined via Luminex screening to identify antibodies towards paternally inherited HLA. (**b**) Mean fluorescence intensity (MFI) of individual FSAs. (**c**) Total MFI of FSAs for each participant. Bars represent median and error bars interquartile range.

Umbilical cord tissue matched to maternal plasma was available for HLA genotype analysis in all eight antibody-positive healthy controls and three women with a diagnosis of CHI in a previous pregnancy. Fetal DNA was unavailable in the remainder of pregnancies from women with previous CHI as two participants had delivered at another centre, and one placenta had not been sent for research. FSAs were evident in five of eight (62.5%) anti-HLA antibody positive control pregnancies compared to all three cases of previous CHI with available fetal DNA (100%) (Fig. 5). There was no statistically significant difference in the number of FSAs in healthy controls compared to cases of previous CHI (2 [IQR 0–3.8] vs 5 [1.0–6.0], respectively) (Fig. 5a). Furthermore, in participants diagnosed with CHI there was no difference in individual MFI value compared to those found in control pregnancies (23,884 [IQR 4723–28628] vs 17,881 [IQR 8345–26500], respectively) (Fig. 5b). Two of three participants with previous CHI exhibited high total MFI of FSAs, though overall there was no statistically significant difference compared to healthy controls (median 108,810 [IQR 3519- 111520] versus 62,132 [IQR 41067-65662], respectively) (Fig. 5d). FSA specificities are listed in Supplementary Table 1.

Using the MFI values of FSAs, the results of FXCM and CDC crossmatch were predicted (Table 2). In one case of previous CHI (33.3%), the B cell FXCM result was equivocal. There were no significant differences in predicted T or B cell positivity for either FXCM or CDC crossmatch in healthy controls or women with a history of CHI.

**Table 2:** Crossmatch prediction results of participants with fetal specific antibodies in healthy control pregnancies and those with a previous diagnosis of chronic histiocytic intervillositis (CHI).

		FCXM		CDC	
	*N*	Positive T cell	Positive B cell	Positive T cell	Positive B cell
Control	5	4 (80.0%)	5 (100%)	4 (80.0%)	4 (80.0%)
Previous CHI	3	2 (66.7%)	2 (66.7%)	2 (66.7%)	2 (66.7%)

*CDC* complement-dependent cytotoxicity, *FXCM* flow cytometry crossmatch. Results predicted via the formula composed by Flynn et al. ^24^. For one participant with a history of CHI, the B cell FXCM result was equivocal, meaning a reliable result could not be established.

## Discussion

Currently, the pathophysiology of CHI is incompletely understood, though pathological similarities shared with rejected allografts, its high recurrence rate, and reported association with maternal autoimmune disease have resulted in the hypothesis that it is a disorder of maternal anti-fetal rejection^10–13,17,20^. Individual case reports on CHI have also described the presence of elevated maternal anti-HLA antibody levels and increased HLA expression at the maternal–fetal interface^15,16^. Despite this evidence, extensive transplant-style crossmatching has yet to be applied to cases of CHI in a more robust case–control study design. Here, virtual crossmatching was used to determine whether CHI resembles organ rejection with respect to C4d deposition and the presence of antibodies directed towards fetal HLA. Placental HLA expression was also investigated to determine whether this is altered in a larger cohort of CHI cases.

C4d deposition has been described in several reports of placentas with CHI^15,17,25^, reminiscent of the appearance of a rejected kidney biopsy^26,27^. Critically, these studies did not compare C4d deposition in index cases of CHI to healthy controls. Here, we found no differences in placental C4d staining between CHI and healthy pregnancies, in part due to the wide variation in the amounts of staining seen in each group. In common with our study, earlier studies of placentas from women with systemic lupus erythematosus (SLE) and antiphospholipid syndrome reported that C4d deposition in healthy controls varies from no detectable staining to a small amount around the apical surface of the syncytiotrophoblast, similar to the staining pattern observed here^28,29^. However, the C4d staining in healthy controls appears elevated compared to previous investigations; it should be noted that the criteria used to grade C4d deposition varies between studies, limiting comparability. Here we found moderate interobserver agreement (κ = 0.43), indicating a degree of subjectivity, which is consistent with wider issues reported in the diagnosis and grading of inflammation in CHI. Specifically, Reus et al.^16^ stated a similar Kappa value of 0.54 for interobserver variability in grading of CHI lesions. Therefore, less subjective methods to grade inflammatory features in CHI need to be developed, for example using computerised quantification or proteomic analyses. In addition, other pathological features e.g., elevated fibrin deposition are apparent in some, but not all placentas with CHI, so C4d likewise might also be exaggerated in specific cases. This is supported by our finding that there was no relationship between CHI severity and C4d deposition. Terry et al.^30^ also stipulated that CHI is “a unique inflammatory process that arises in a permissive environment after exposure to an appropriate trigger”. If this is the case, it may be plausible that complement activation in CHI acts in combination with other aberrant inflammatory processes to cause pathology, whereas in healthy pregnancy its activity is limited, e.g. by complement regulatory factors richly expressed on the placenta^31^. Further characterisation of immunoregulatory molecules in CHI in future may help to confirm or refute this hypothesis.

The proportion of participants with anti-HLA antibodies did not differ significantly between controls and women with previous CHI, at 47.1% versus 40.0%, respectively. Additionally, there were no differences in the proportion of positive predicted crossmatch results, %cRF value, the number of FSAs or their MFI. With regard to anti-HLA antibodies, pregnancy is considered to be both the most common and strongest sensitising event, and anti-HLA positivity is reported in 29–49% of parous women^32–35^. The mechanism of HLA sensitisation in healthy pregnancy appears to be as a result of extracellular vesicles released from the placenta into the maternal circulation and interaction with fetal cells at delivery providing a source of fetal antigen^36–38^. Previous studies of a total of six women with CHI, reported maternal anti-HLA antibody positivity to be as high as 75%, though only one investigated a control group, consisting of seven participants^15,16^. In contrast, evidence from this more robust study instead suggests that anti-HLA antibody sensitisation in women with previous pregnancies complicated by CHI is comparable to parous healthy controls. Nevertheless, despite this lack of difference in HLA antibody profiles between healthy pregnancies and those with a history of CHI, index cases within our cohort did exhibit elevated HLA-Class II at the maternal–fetal interface. It is well recognised that in healthy pregnancy the semi-allogeneic human placenta is tolerated by the maternal immune system, largely thanks to downregulation of HLA by the villous trophoblast^2^. With this in mind, it is reasonable to hypothesise that an altered HLA profile in placentas with CHI may mean that anti-HLA antibodies are more relevant in these pregnancies, even if the level of sensitisation is similar to healthy controls. As HLA-Class II expression in placentas from index cases of CHI within our cohort showed a positive relationship with the extent of C4d deposition and maternal CD68^+^ macrophage infiltration, it is tempting to speculate that binding of anti-HLA antibodies to the trophoblast may occur in severe cases of CHI, particularly as HLA-Class II expression was observed in proximity to areas of intervillous macrophage infiltration. Further investigation is required to determine how these inflammatory features may be related, for example it would be worthwhile to determine whether C4d deposition is localised to areas where HLA is upregulated on the trophoblast, suggesting evidence of antibody binding at the maternal–fetal interface. Our finding of HLA-Class II on the syncytiotrophoblast surface aligns with an observation made in Benachi et al.’s^15^ study of two placentas with CHI, although importantly no significant difference in overall HLA-Class I expression was found here after semi-quantitative grading in a larger cohort. It should be noted that within our study, interobserver agreement was only fair, again emphasising the need for less subjective measurement of inflammatory features in CHI. In a similar study into villitis of unknown etiology, a related inflammatory placental condition, Enninga et al. analysed gene expression via microarray and found altered expression of Class-II mRNA and upregulation of pathways associated with graft rejection. Use of similar methods in CHI may therefore provide better insight into its pathophysiology and reduce subjectivity associated with manual grading.

As well as anti-HLA antibodies, CHI has been associated with maternal autoimmune disease and fetal and neonatal alloimmune thrombocytopenia (FNAIT)^11,12,39^. However, the link between autoantibodies and development of CHI remains unclear and reported rates of autoimmune disease in women with CHI vary widely, from 12 to 58%^11,12,18^. In studies of women with antiphospholipid syndrome, use of hydroxychloroquine was associated with reduced titres of autoantibodies which bind to trophoblast in vitro and initiate release of pro-inflammatory cytokines^40,41^. In subsequent pregnancies following a diagnosis of CHI, hydroxychloroquine and other immunomodulatory medications e.g. prednisolone are often prescribed with an aim to reduce the maternal inflammatory response^42^. It is therefore conceivable that maternal antibodies may have been present in index pregnancies affected by CHI but without accompanying C4d deposition, similar to a phenomenon termed as C4d-negative rejection, recently acknowledged in antibody-mediated rejection wherein complement is not detected in graft biopsies^27^. Applying the same experimental method in index CHI pregnancies is limited by the fact that maternal plasma taken during pregnancy from these cases would not be available as diagnosis is made after delivery. An alternative method would be to directly apply anti-human IgG or IgM to stored placental tissue from these cases to highlight any bound anti-placental antibody, similar to that used in the diagnosis of lupus via skin biopsies, known as the lupus band test^43^. Obtaining plasma from women with antiphospholipid syndrome would also likely serve as a suitable positive control to ensure antibodies are detected via the assay. As CHI has been observed in cases of FNAIT and autoimmune disease, it is also plausible that it may occur as a result of a variety of antibodies which are capable of binding to antigens expressed on the placenta, rather than HLA alone. This may help to explain why some, but not all participants with CHI tested positive for anti-HLA antibodies within this study.

As a diagnosis of CHI is only made after an affected pregnancy, the plasma analysed in this study was limited to subsequent pregnancies, all of which were receiving a form of thromboprophylactic and immunomodulatory treatment that may have prevented CHI recurrence^18^. Following solid organ transplantation, prednisolone is used routinely as maintenance immunosuppression to prolong graft survival. It is therefore possible that any anti-HLA antibodies which may have been present in previous pregnancies were reduced as a result of medication. This limitation extends beyond this study and into research on CHI as a whole, as plasma is rarely collected at the time of index pregnancies and women are increasingly beginning to take immunosuppressive medications even before conception, making screening for maternal antibodies in this period and pregnancy before treatment difficult. Furthermore, ethical issues surrounding randomised control trials using placebo or less effective medications in women with multiple previous poor pregnancy outcomes means that obtaining plasma from untreated pregnant women is unlikely. To address the confounding factor of medication, it may be worthwhile in future to obtain ethical permission to obtain blood for anti-HLA antibodies outside of pregnancy, before immunomodulatory treatment has commenced. Preliminary data from two women with CHI suggested that fetal-specific anti-HLA antibodies are stable for up to 17 months, further justifying the use of sampling following the first affected pregnancy^15^. The previously referenced study identifying CHI in 40.7% of placentas from women with FNAIT tested for antibodies in plasma at four separate timepoints from 16–20 weeks’ gestation^39^. Antibody-positive women were defined as those with a positive screen in at least one of four samples, as antibodies may be transient. In contrast, plasma from only one time point during gestation was available for analysis here, which could have negatively affected Luminex results. Thus, further investigations using maternal plasma sampled as soon as possible after the index case and longitudinal studies may give more accurate insight into the role of anti-HLA antibodies and how they fluctuate throughout gestation.

To date, this study represents the largest attempt to apply crossmatching techniques in samples from women with a previous diagnosis of CHI. In collaboration with an accredited Transplantation Laboratory, extensive anti-HLA antibody screening was undertaken to a high standard as used in the clinical setting to determine donor-recipient suitability pre-transplant. In the majority of cases, matched placental tissue and fetal DNA was also available, which allowed any fetal-directed antibodies to be identified and for the prediction of crossmatch results. Though no differences were found, this may be a result of immunomodulatory therapy taken by participants, and sample size was limited for these variables as only three and five cases of CHI and controls had antibodies toward their fetus, respectively. As CHI is a rare disease, increasing involvement of affected women into research studies and facilitating collaboration between centres specialising in its treatment will be essential to provide a larger sample size to more thoroughly investigate the specific relevance of antibodies which may be directed towards fetal HLA. As CHI has been hypothesised as an inappropriate immune response toward fetal HLA inherited from the father, both previous studies to investigate anti-HLA antibodies had paternal DNA available for genotyping^15,16^. In this study, analysis was restricted to fetal DNA due to the fact that collection of paternal plasma samples was not covered by ethical agreements. This meant that only anti-HLA antibodies directed toward the fetus of the current pregnancy could be identified, as opposed to other specificities which may have been present in past pregnancies with CHI. Thus, future studies investigating the immune aetiology of CHI should include collection and analysis of paternal DNA.

Though CHI has been likened to maternal rejection of the placenta, evidence of increased C4d deposition and increased maternal anti-fetal antibodies could not be confirmed. Despite this, we have described for the first time in a relatively large cohort of CHI cases that HLA-Class II expression is upregulated at the maternal–fetal interface. This leads us to hypothesise that anti-HLA antibodies may hold more relevance in the pathophysiology of CHI compared to healthy pregnancies where trophoblast HLA expression is restricted. What remains to be answered is whether increased HLA expression in CHI is causative, or a secondary response to placental inflammation. Due to the heterogeneity in the degree of HLA-Class II upregulation within our cohort, we argue that the latter is more likely, however the initial inflammatory trigger in CHI is yet to be identified. In investigating the role of HLA in CHI, treatment using immunosuppressive medication in subsequent pregnancies and subjectivity in the grading of inflammation are likely confounding factors. Further study using transcriptomic analysis of inflammatory pathways and detection of antibodies after an initial diagnosis of CHI is therefore required, both to identify its cause and potential therapeutic targets.

### Supplementary Information

Supplementary Figure 1.

Supplementary Table 1.

Supplementary Legends.

## Data Availability

The datasets generated during and/or analysed during the current study are available from the corresponding author on reasonable request.
