# Supplementary figures and images for: Virtual crossmatching reveals upregulation of placental HLA-Class II in chronic histiocytic intervillositis
Source: Sci Rep. 2024 Aug 12;14:18714. doi: 10.1038/s41598-024-69315-5 (PMC11319473; doi:10.1038/s41598-024-69315-5)

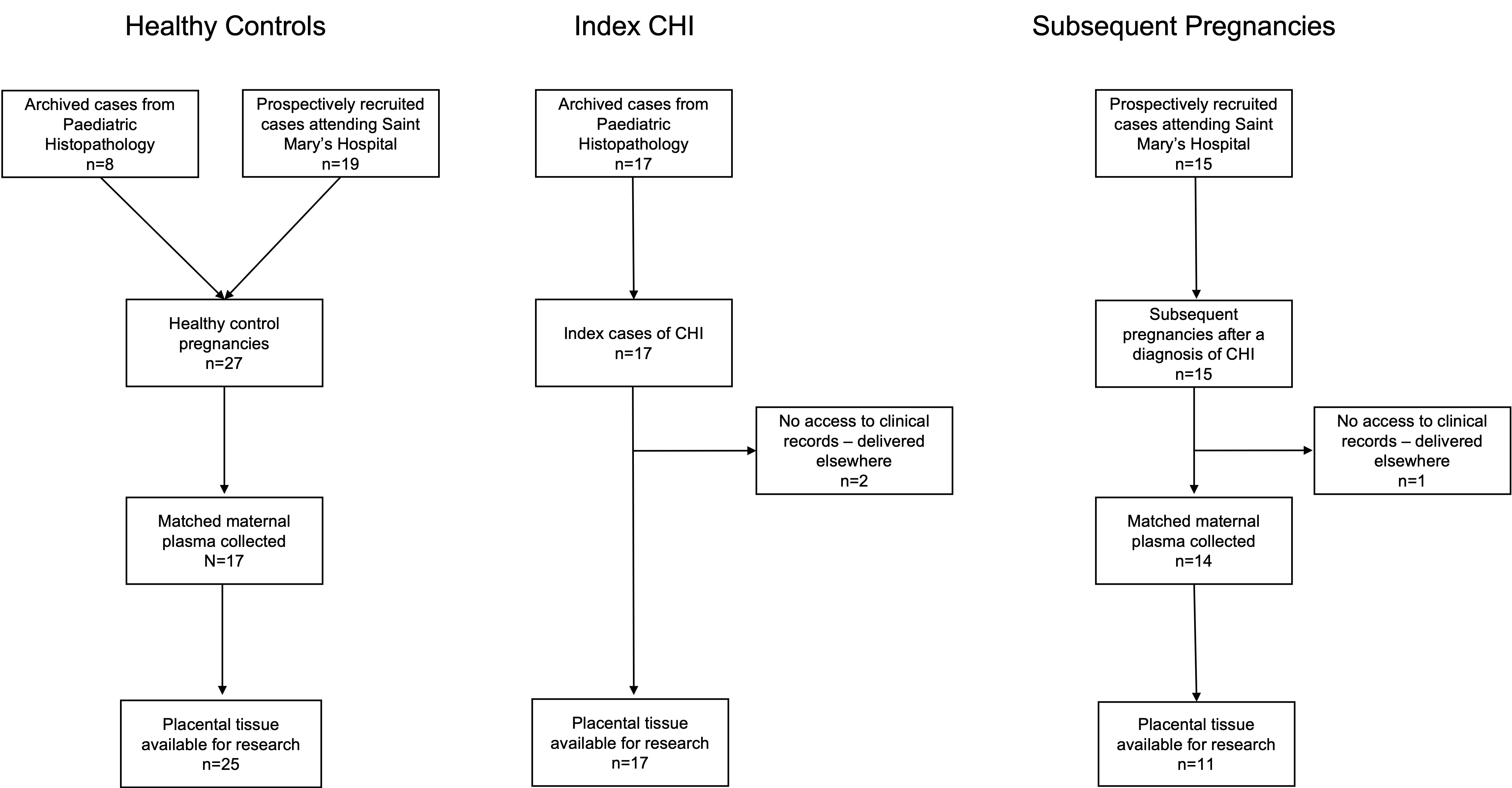

Supplement: Supplementary file 1 — Supplementary Figure 1. [file 41598_2024_69315_MOESM1_ESM.png]
