# Supplementary material for: Virtual crossmatching reveals upregulation of placental HLA-Class II in chronic histiocytic intervillositis
Source: Sci Rep. 2024 Aug 12;14:18714. doi: 10.1038/s41598-024-69315-5 (PMC11319473; doi:10.1038/s41598-024-69315-5)
Supplement: Supplementary file 2 — Supplementary Table 1. [file 41598_2024_69315_MOESM2_ESM.docx]

## Supplementary Data

**Supplementary Table 1. Fetal-specific anti-HLA antibody (FSA) specificities in healthy control pregnancies and those following a previous diagnosis of chronic histiocytic intervillositis (CHI).**

|  | **FSA Specificity** | | | | | |
| --- | --- | --- | --- | --- | --- | --- |
|  | **Class I** | | | **Class II** | | |
| **Control** | HLA-A | HLA-B | HLA-C | HLA-DP | HLA-DQ | HLA-DR |
| Case 1 |  |  |  |  |  | + |
| Case 2 | + | + |  |  | + | + |
| Case 3 |  | + | + |  |  | + |
| Case 4 | + | + |  |  | + | + |
| Case 5 | + |  |  |  |  | + |
| **Previous CHI** |  |  |  |  |  |  |
| Case 1 |  | + | + | + |  | + |
| Case 2 |  |  |  |  | + |  |
| Case 3 | + | + | + |  | + | + |

Antibody positivity against particular HLAs is indicated with ‘+’.
