# Supplementary material for: Virtual crossmatching reveals upregulation of placental HLA-Class II in chronic histiocytic intervillositis
Source: Sci Rep. 2024 Aug 12;14:18714. doi: 10.1038/s41598-024-69315-5 (PMC11319473; doi:10.1038/s41598-024-69315-5)
Supplement: Supplementary file 3 — Supplementary Legends. [file 41598_2024_69315_MOESM3_ESM.docx]

**Supplementary Figure 1** Study recruitment and sample collection flowchart. Index CHI refers to the first pregnancy to be diagnosed with chronic histiocytic intervillositis (CHI).
